# Hemorrhagic Disease of the Newborn as a Consequence of Vitamin K Refusal Due to Language Barrier

**DOI:** 10.7759/cureus.57065

**Published:** 2024-03-27

**Authors:** Moustafa Elsebey, Vidya Nandlal, Florentina Litra

**Affiliations:** 1 Department of Pediatrics, University of Florida, Pensacola, USA; 2 Department of Pediatrics, Ascension Sacred Heart Pensacola, Pensacola, USA

**Keywords:** language interpreter, communication, hemorrhage, vitamin k deficiency, vitamin k, newborn

## Abstract

Newborns are prone to hemorrhagic disease due to vitamin K deficiency for multiple reasons, including vitamin K absence in breast milk and formula preparation, sterile gut with limited absorption, and lack of placental transfer.

Despite the importance of vitamin K administration at birth in preventing hemorrhagic disease in infants, some parents still refuse administration to their newborns. One of the unexpected but preventable reasons is the language barrier related to special dialects, resulting in misunderstanding the benefits of vitamin K administration and complications related to vitamin K deficiency.

We present a case of hemorrhagic disease of the newborn due to vitamin K deficiency following the parental refusal of postnatal prophylactic vitamin K due to a language barrier that resulted in miscommunication. Although appropriate education was provided to the family via Spanish interpreter as requested, it was later revealed that the family was speaking a special dialect, and they did not fully understand the importance of vitamin K prophylaxis. The patient experienced intracranial hemorrhage with full recovery after treatment and surgical intervention. Upon reviewing the case, the parents were speaking a rare dialect of Spanish, that was not known to the Spanish medical interpreters. A combination of a Spanish medical translator and a family friend was necessary for effective communication with the family.

## Introduction

Vitamin K is a crucial cofactor for the activation of coagulation factors VII, IX, X, and prothrombin as well as protein C and S as it is required for γ-glutamyl carboxylase enzymatic activity that activates the gamma-carboxylation of specific glutamic acid residues in a subclass of proteins within the hepatic cells [1].

Newborn infants are vulnerable to vitamin K deficiency due to lack of transplacental transfer of vitamin K during pregnancy, breast milk, and formula preparation deficient in vitamin K, and limited gut absorption due to lack of gut bacteria. Exogenous vitamin K is given for the prevention of bleeding immediately after birth [2]. Prophylactic administration of vitamin K is recommended for all newborns to prevent vitamin K deficiency bleeding (VKDB; previously referred to as hemorrhagic disease of the newborn).

In the United States, routine postnatal administration of vitamin K has made VKDB rare. The frequency of VKDB varies from 0.25% to 1.7% in infants not receiving vitamin K prophylaxis in the first week of life. The risk of VKDB decreased significantly from 1,700 per 100,000 infants if vitamin K is not given, to 1 per 100,000 infants when vitamin K is provided [3].

We advocate for providers to educate parents, provide educational resources, emphasize parental understanding of the safety and benefits of vitamin K administration, and explain the consequences and complications due to lack of administration.

## Case presentation

A five-week-old previously healthy full-term infant presented to the Emergency Department (ED) with non-bloody non-bilious vomiting, loose, non-bloody diarrhea, tactile fever, and increased fussiness for one day. The child was exclusively breastfed, and his mother was the only known sick contact with a mild upper respiratory infection. Pregnancy was unremarkable. The infant was born full term, without complications, and received hepatitis B vaccine at birth. He had an unremarkable stay in the newborn nursery and was discharged after two days of life. A medical translator service was used due to a language barrier; however, the mother’s friend was helping to translate portions of the history as the family was speaking a dialect unrecognized by the medical translator. The physician documented discussions with the family about vitamin K through the Spanish medical translator, and ordered the vitamin K; however, they eventually declined the dose, when the nurse attempted to administer it.

At presentation to ED, the patient appeared ill, but with vital signs normal for his age: temperature 36.6 C; blood pressure 96/49 mm Hg; heart rate 145 beats/min; respiratory rate 42 breaths/min; oxygen saturation 100% on room air. His physical exam was significant for signs of mild dehydration including dry mucous membranes and mildly sunken eyes but had full anterior fontanel, otherwise unremarkable. Initial laboratory data (Table 1) showed normocytic normochromic anemia (hemoglobin (Hgb): 8.1 mg/dl) with thrombocytosis (587 K/uL), hyponatremia (127mg/100ml), mild transaminitis (alanine transaminase (ALT) 73 Intl Units/L), aspartate transaminase (AST) (69 Intl Units/L), hyperbilirubinemia (total bilirubin 4.1 mg/100ml), mildly elevated procalcitonin (0.14 ng/mL) with normal C-reactive protein (CRP) (0.60 mg/dL), mildly elevated lactate (3.1 mmol/L) with metabolic acidosis. Human rhino-enterovirus was detected in the respiratory viral pathogens panel. Blood and urine cultures were obtained, lumbar puncture (LP) yielded light pink cerebrospinal fluid (RBCs 2,810/cumm, WBCs 5/cumm), and empirical antibiotics (ampicillin and gentamycin) were administered intravenously (IV), in sepsis doses.

**Table 1:** Initial laboratory data

Diagnostic lab	Patient value	Range
Hemoglobin	8.1 mg/dl	10-14 mg/dl
Platelet count	587 K/uL	200-400 K/uL
Serum Sodium	127 mg/100ml	139-146 mg/100ml
Alanine transaminase (ALT)	73 Intl Units/L	11-33 Intl Units/L
Aspartate transaminase (AST)	69 Intl Units/L	20-67 Intl Units/L
Total bilirubin	4.1 mg/100ml	0.1-0.7 mg/100ml
Procalcitonin	0.14 ng/mL	0.0-0.1 ng/mL
C-reactive protein (CRP)	0.60 mg/dL	≤ 0.90 mg/dL
Serum lactate	3.1 mmol/L	0.9-2.6 mmol/L
Respiratory viral pathogens panel (RPP)	Human rhino-enterovirus	Negative
Cerebrospinal fluid RBCs	2,810/cumm	0-5/cumm
Cerebrospinal fluid WBCs	5/cumm	0-6/cumm
RBCs: Red Blood Cells, WBCs: White Blood Cells		

Within a few hours after being admitted to the medical unit, the patient had a change in his mental status with irritability, and the left pupil was fixed and dilated, non-reactive to light. On the exam, we also noted bleeding from his IV site on the right forearm and the LP site. Repeat labs (Table 2) showed a large drop in Hgb (4.4 mg/dl) with worsening metabolic acidosis and increased lactate (10 mmol/L). The patient received two normal saline boluses and was taken for stat computerized tomography (CT) brain without contrast, but the patient experienced respiratory distress and bradycardia during scanning and was brought to the Pediatric Intensive Care Unit (PICU) emergently.

**Table 2:** Repeat laboratory data

Diagnostic lab	Patient value	Range
Hemoglobin	4.4 mg/dl	10-14 mg/dl
Serum Lactate	10 mmol/L	0.9-2.6 mmol/L

**Figure 1:**
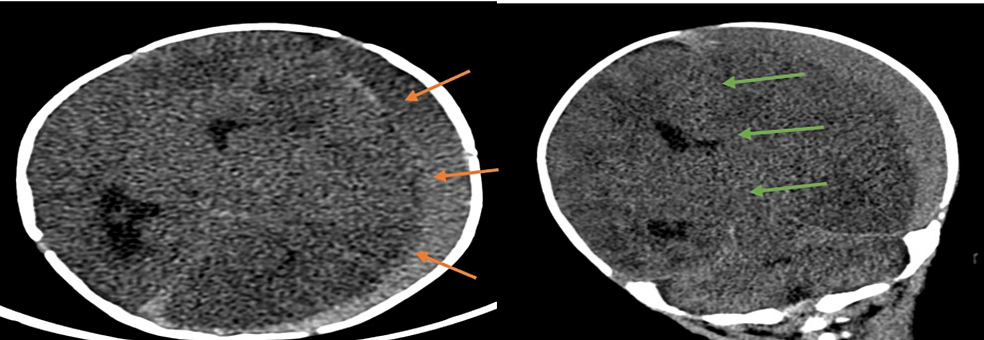
Noncontrast CT brain after initial presentation showing large mixed attenuation subdural hemorrhage involving the left frontal, parietal, temporal and occipital regions (orange arrows) with marked mass effect on the underlying brain parenchyma with 1.4 cm of left-to-right midline shift and asymmetry of the lateral ventricles (green arrows).

In the PICU, the patient was intubated and mechanically ventilated. Type and cross match and coagulation profile drawn, and unmatched O negative 5 ml/kg packed red blood cells (PRBCs) and 10 ml/kg fresh frozen plasma (FFP) emergently given for the treatment of hemorrhagic shock and uncontrollable bleeding, as the CT revealed large mixed attenuation subdural hemorrhage (SDH) with 1.4 cm of left-to-right midline shift and asymmetry of the lateral ventricles (Figure 1). Upon reviewing the newborn discharge summary, it was noted that the patient's parents refused to give him postnatal prophylactic vitamin K. Per further history obtained with the mother and translator, it was found out that although the newborn physician explained the benefits of vitamin K via translator and ordered the dose, the parents eventually changed their minds by the time of nurse came to administer it, due to lack of clear understanding of the potentially devastating consequences of the vitamin K deficiency.

Emergently, the patient received vitamin K and prothrombin complex concentrate (Kcentra®) while awaiting the coagulation profile results that later showed profound coagulopathy (prothrombin Time (PT), > 300s; international normalized ratio (INR), > 4; and partial thromboplastin time (PTT), > 300s with normal fibrinogen level). The coagulation panel normalized within two hours of emergent treatment with FFP, vitamin K, and Kcentra (Table 3).

**Table 3:** Pre- and post-treatment coagulation profile

Diagnostic lab	Pre-treatment	Post-treatment	Range
Prothrombin Time (PT)	> 300 seconds	14.3 seconds	11.6-15 seconds
International Normalized Ratio (INR)	> 4	1.1	≤ 1.1
Partial Thromboplastin Time (PTT)	> 300 seconds	26.9 seconds	23-40 seconds
Fibrinogen	345 mg/dL	306 mg/dL	200-400 mg/dL

**Figure 2:**
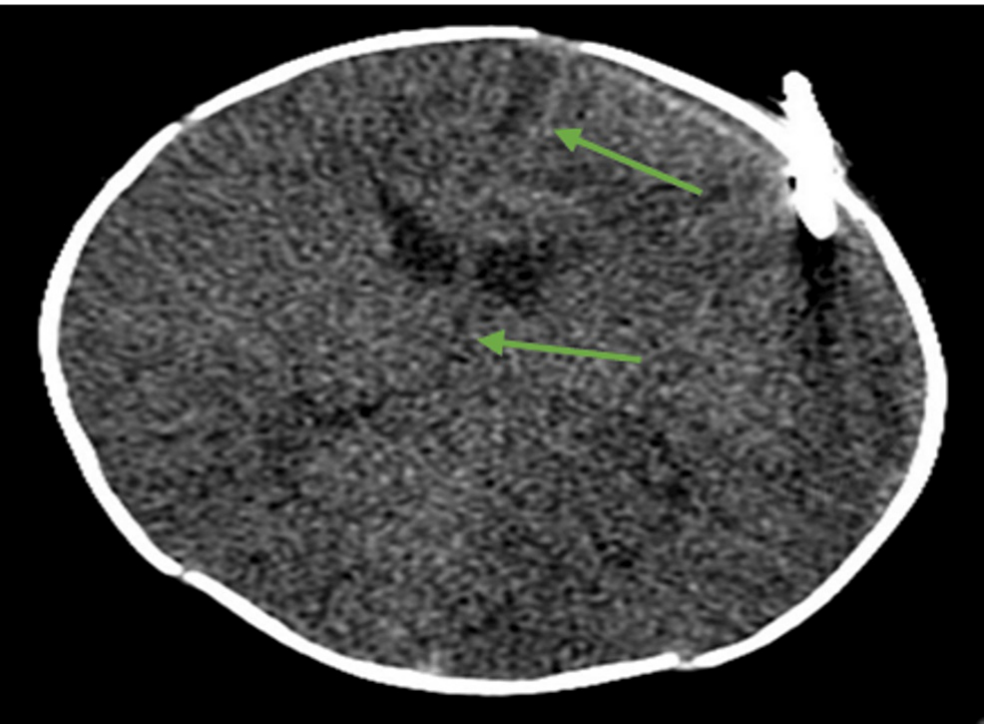
Noncontrast CT brain after interval placement of left-sided subdural drain with significant interval reduction in the volume of the subdural hematoma and significant improvement in mass effect and midline shift measuring approximately 7-8 mm (green arrows).

**Figure 3:**
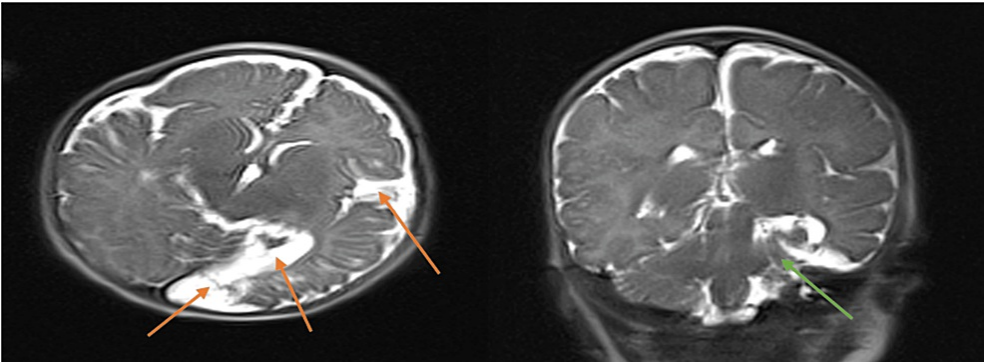
T2, diffusion-weighted magnetic resonance imaging, four weeks after discharge, showing evolution of the left cerebral infarction predominantly involving the left temporal and left occipital lobe with gliosis in this region (green arrow) and increased prominence of the surrounding extra-axial fluid spaces and adjacent left lateral ventricle most likely representing ex vacuo phenomenon. There is no mass effect, midline shift, herniation, or edema. Cerebral volume loss in the left temporal occipital lobes (orange arrows).

Neurosurgery was consulted and an emergent decompression of left SDH was done at the bedside in the PICU then the patient was taken to the operation room (OR) for an emergent left craniotomy, evacuation of remaining hematoma, and drain placement, the patient required extra 60 mL packed red blood cells transfused during neurosurgical operation. Post-operative CT brain was done and showed significant improvement in mass effect and midline shift compared to prior (Figure 2). Prophylactic anti-seizure medication was initiated. At 48 hours, the patient was extubated to room air, the subdural drain was removed, and antibiotics were discontinued as all cultures were negative. Bedside swallow evaluation by speech therapy was reassuring, and he was cleared to breastfeed. The patient fully recovered, prophylactic anti-seizure medication was stopped, and he was discharged home on hospital day 7. Follow-up magnetic resonance imaging (MRI) brain four weeks after discharge revealed continued resolution of the left SDH (Figure 3).

## Discussion

We would like to draw attention to an alarming case that highlights the grave consequences of parental refusal to administer prophylactic vitamin K to newborns. The condition known as late-onset VKDB can result in intracranial hemorrhage as the primary symptom in as many as 30-60% of affected infants, with a mortality rate of 14% associated with this devastating condition. It is vital for parents to understand the importance of prophylactic measures in preventing such tragic outcomes [2].

In this case, the cause of acute intracranial bleeding and coagulopathy was identified as VKDB, supported by the history of parental refusal, laboratory data, and normalization of the coagulation profile after administration of vitamin K and FFP [4]. However, intracranial hemorrhage occurred before VKDB diagnosis. Perinatal preventive practices are critical to avoid the morbidity and mortality of late-onset VKDB.

Late-onset VKDB was first identified in 1977 in a study of 93 Thai infants who were affected [5]. It is a condition where bleeding occurs in infants between two weeks and six months of age. It typically occurs most frequently between the third and eighth weeks of life [6]. Studies have reported varying mean ages for infants with late VKDB. One study found that the mean age was 10.3 weeks (range: 7-20 weeks) [2], while others reported that the mean age was between three and seven weeks [7]. Another study reported that the peak age for late VKDB was four weeks, with 79% of affected infants being between three and seven weeks old [8].

Although later onset hemorrhagic disease of the newborn is a rare incidence, it is a highly preventable disease with potentially extreme consequences. As we saw with this patient, an otherwise healthy newborn went from stable discharge to critical illness a few weeks after birth. The parents were unclear of the role of vitamin K in preventing such disease, and the severe costs that potentially could impact their child’s health. In this case, the language barrier and dialect difference between the translating services used and the native language of the patient’s family were a large contributing factor in the refusal of vitamin K.

Over the past decade, global migration rates have increased by 50%, with a significant number of migrants settling in the United States [9]. According to the U.S. Census Bureau, about 25 million people in the country have limited English proficiency (LEP). This highlights the importance of effective communication with such patients to ensure successful care [10]. A study shows that miscommunication was responsible for 59% of serious adverse events reported to the Joint Commission [10].

Studies have revealed that language barriers between healthcare providers and patients can result in a higher incidence of negative outcomes [11]. In hospital-based research, it was observed that 49.1% of patients who had limited proficiency in the English language experienced physical harm as opposed to 29.5% of those who spoke English fluently [12].

Professional medical interpreters can help overcome language barriers in healthcare settings, but they cannot eliminate patient safety risks. Furthermore, relying on interpreting services may negatively impact the physician-patient relationship, as it is not as effective or satisfying as direct communication with a healthcare professional [13].

Health disparities, which may arise from unequal treatment due to language barriers, can result in unequal access to healthcare and ultimately lead to a disparity in health outcomes [14]. Studies have indicated that patients who are unable to speak the local language face a disadvantage when it comes to accessing healthcare services [15]. Furthermore, patients who experience language barriers have been shown to have worse health outcomes than those who do speak the local language [16].

Many parents tend to rely on internet search engines and various social media platforms to gain knowledge about critical healthcare scenarios. However, the information they find may not always be accurate or trustworthy, leading to potential misinterpretation and confusion [17,18].

In terms of the most appropriate use of vitamin K prophylaxis, the American Academy of Pediatrics (AAP) recommends all newborn infants receive a single intramuscular (IM) dose of 0.5 to 1.0 mg of vitamin K [19]. Although oral vitamin K has been shown to be effective in preventing the classic form of VKDB, multiple reports have cited late-occurring VKDB in countries that recommend oral prophylaxis via a single oral dose of vitamin K at 2 mg per dose [19,20]. Several European countries that primarily used oral vitamin K prophylaxis to prevent this disease have increased the dosing schedule from a single dose after birth to dosing on a schedule to effectively decrease the incidence of the disease [20]. For example, in Switzerland, the recommended schedule from surveillance studies recommended three oral doses of 2 mg of vitamin K to be given at day 1, day 4, and week 4 of life to significantly lower the rate of late VKDB to 0.87 per 100,000 [19]. In that same surveillance study, one of the main risk factors reported again was found to be “parental refusal of any prophylaxis,” along with authors concluding efficacy decreased when given via oral route due to poor parental compliance with the regimen and variable drug absorption [19].

## Conclusions

Providers should advocate for vulnerable children to limit the preventable complications from vitamin K deficiency due to parental refusal, based on parents’ preference, religious beliefs, or language barrier.

Providers should educate parents and emphasize parental understanding of the safety and benefits of vitamin K administration and explain the potentially severe hemorrhagic consequences related to vitamin K deficiency. Many hospitals now ask parents to sign an opt-out form, stating that they fully understand the risks and potentially devastating complications of their decision. When the language barrier is a concern, the responsibility falls on the healthcare staff and they should find accessible tool solutions to facilitate proper communication with the parents as the patient’s life may depend on clear communication. An especially challenging circumstance is when parents speak a special dialect, and they may not readily disclose that to the medical team. A combination of a certified medical translator and a family member may be more efficient than the translator alone in this unique situation.
